# Comparative transcriptome analysis of *pectoralis major* muscles affected by white striping, wooden breast and spaghetti meat in male and female broiler chickens

**DOI:** 10.1186/s12864-025-11963-6

**Published:** 2025-08-21

**Authors:** Marija Bošković Cabrol, Marianna Pauletto, Mery Giantin, Mauro Dacasto, Gerolamo Xiccato, Angela Trocino

**Affiliations:** 1 Department of Agronomy, Food, Natural Resources, Animals and Environment (DAFNAE), University of Padova, Legnaro, Padova, 35020 Italy; 2 Department of Comparative Biomedicine and Food Science (BCA), University of Padova, Legnaro, Padova, 35020 Italy; 3 Department of Food Hygiene and Technology, Faculty of Veterinary Medicine, University of Belgrade, Belgrade, 11000 Serbia

**Keywords:** Myopathy, Chicken breast, RNA sequencing, Sex, Differentially expressed genes, Etiopathogenesis

## Abstract

**Background:**

Growth-related myopathies, including white striping (WS), wooden breast (WB), and spaghetti meat (SM), compromise broiler meat quality, causing significant economic losses. Although these myopathies share some histological features, their molecular mechanisms remain incompletely understood, particularly regarding sex-specific differences. This study aimed to compare transcriptomic profiles of normal and defective *pectoralis major* muscles to identify biological pathways underlying male and female myopathies. Transcriptomic analysis was performed on RNA-seq data from *pectoralis major* muscles of male and female Ross 308 broilers. Then, differentially expressed genes (DEGs) and enriched pathways were identified using edgeR and functional annotation tools.

**Results:**

SM samples exhibited the most extensive transcriptional alterations, particularly in males, with significant disruption of pathways related to hypoxia, energy metabolism, calcium signaling, and extracellular matrix remodeling. Compared to normal meat, WB meat demonstrated moderate molecular changes, while WS meat showed minimal transcriptomic impact. Males displayed pronounced metabolic dysregulation and increased activation of pathways associated with inflammation, fibrosis, and vascular remodeling compared to females, where transcriptional changes were generally less pronounced across all myopathies. Shared pathways among myopathies included oxidative phosphorylation, cytoskeletal organization, and inflammatory responses, though their expression patterns varied between sexes and conditions.

**Conclusions:**

This study highlights marked sex-specific differences in molecular responses to WS, WB, and SM, with males exhibiting more pronounced transcriptomic alterations. These findings underscore the importance of sex-specific approaches to mitigate the economic and welfare impacts of these myopathies in broiler production.

**Supplementary Information:**

The online version contains supplementary material available at 10.1186/s12864-025-11963-6.

## Background

Spaghetti meat (SM), white striping (WS), and wooden breast (WB) are myopathies afflicting broiler chickens and are characterized by distinct muscular abnormalities, yet sharing histological features, which suggests similar aetiology [1–3]. Although defective meat does not pose a public safety risk [4, 5], the substantial economic losses [6, 7], primarily attributable to the compromised quality and disposal of such meat, emphasize the need for effective mitigation strategies.

Understanding the underlying causes of muscle abnormalities in poultry is crucial for developing a comprehensive strategy to mitigate these issues. Fast growth rate, increased body weight, and high breast meat weight and yield have been identified as the key factors responsible for the increased occurrence and severity of myopathies [8–10]. Regarding genetics, low to moderate heritability has been reported for WS (h^2^ = 0.65) [9], whereas a low heritability was found for WB (h^2^ < 0.01) [11]. The sex predisposition for myopathies remains unclear, although some studies suggest that male broilers are more susceptible to WB [12–14], while SM incidence is found to be higher for female birds [14–16].

Few transcriptomic studies suggest WS and WB have similar pathogenesis, including hypoxia and oxidative stress, unsuccessful angiogenesis, leading to degeneration and apoptosis, inflammation, regeneration, lipidosis, and fibrosis within the muscle [17–20]. Different expressions of genes associated with energy metabolism [21] and calcium signaling [22] have also been found in meat affected by WS and WB, whereas the data on SM, the most recently discovered myopathy, remains scarce. The recent study of Che et al. [3], the first to compare the transcriptomes of SM and WB, showed that these two myopathies have comparable enriched metabolic pathways and processes.

The transcriptomic profile of myopathies, and especially that of WB myopathy [3, 13], is under extensive investigation, with their occurrence rate differing between males and females. However, to our knowledge no published data have compared the transcriptomes of all three *p. major* myopathies within the two sexes.

Therefore, in the present study, we compared the transcriptome of normal and defective *p. major* muscles to identify possible similarities and differences in the biological mechanisms leading to WS, WB, and SM myopathies in male and female chickens.

## Methods

### Animals and sample collection

The present study was carried out using the *p. major* muscles obtained from our previous experiment. The details about housing, farm management conditions, and diets are described in the companion paper [16] which gave results related to growth traits and meat quality. Briefly, male and female Ross 308 broiler chickens were reared in pens in two rooms of the poultry house of the University of Padova where they were allocated to 12 experimental groups, i.e., 2 temperatures (heat stress- 28 °C and thermoneutral- 20 °C) × 2 sexes (males and females) × 3 diets (containing 0%, 3% or 6% of *C. vulgaris* meal). Birds received feed and water ad libitum. At 42 days of age, chickens were transported to a commercial poultry slaughterhouse, electrically stunned, and immediately slaughtered by severance of the jugular veins according to standard commercial practices. After 2-h chilling, 180 carcasses (5 per pen) were selected based on the average bird live weight and variability of the corresponding pens and transported to the DAFNAE laboratory, University of Padova, and visually evaluated for the presence or absence of WS, WB, and SM [23, 24]. Thus, chickens fed only the control diet were used to sample the available normal or defective *p. major* muscles for the present gene expression analysis, i.e. 6 normal breasts (3 females and 3 males), 6 WS breasts (3 females and 3 males), 5 WB breasts (2 females and 3 males), and 5 SM breasts (3 females and 2 males) were used for this experiment (Supplementary Table 1, Additional file 1). The study had been designed to collect samples of meat with myopathies from animals whose life history was completely known, i.e. animals kept under controlled conditions. However, under the conditions of our study, the rate of myopathies was moderate in the case of WS (25.5%) and SM (18.3%), and low for WB (8.9%) [25], which challenged the total number of animals fed the control diet to be sampled. In fact, myopathy occurrence is not predictable and can vary under different farming conditions. While the low number of samples for some experimental groups (e.g., SM samples from males) can be considered a limitation of the present study, other studies about myopathies have also analysed a limited number of samples for transcriptomics [26–28], mainly because of cost reasons, and none of them faced the three myopathies in the two sexes at the same time.

### Sampling, RNA extraction and sequencing

Meat samples (approximately 1 cm^2^) from the cranial region were excised under RNase-free conditions and stored in RNAlater^®^ reagent (Applied Biosystems, Foster City, CA, USA). Samples were stored at 4 °C overnight and then transferred to −80 °C until RNA extraction.

Total RNA was extracted using the RNAeasy Mini Kit (Qiagen, Hilden, Germany), following the manufacturer’s instructions. Total RNA purity was assessed through a NanoDrop 1000 Spectrophotometer (Thermo Fisher Scientific, Waltham, MA, USA), while total RNA concentration was determined using a Qubit RNA BR (Broad-Range) kit in a Qubit 4.0 Fluorometer (Life Technologies, Carlsbad, CA, USA). RNA quality was assessed using a 4150 TapeStation System (Agilent Technologies, Waldbronn, Germany). All samples had an RNA integrity number > 7. Twenty-eight tagged RNA-seq libraries were prepared using the Novogene NGS RNA Library Prep Set (PT042) and sequenced on an Illumina NovaSeq 6000 instrument at the Novogene Biotechnology (Cambridge, United Kingdom) following a 150-bp paired-end approach.

### RNA‑seq reads processing and mapping

Initial quality control was performed using FastQC software v.0.11.9 [29]. Reads trimming and adapter removal were performed using Trimmomatic (v.0.39) with default parameters [30]. Reads shorter than 36 bp were excluded from the analysis. Residual ribosomal RNAs (rRNAs) were removed using Bowtie2 (v.2.2.9; [31] by mapping the reads against the sequences of ribosomal small and large subunits available in the SILVA database for Bacteria, Archaea and Eukaryotes (https://www.arbsilva.de/no_cache/download/archive/current/Exports/). Survived reads were pseudoaligned to the chicken reference transcriptome (GCA_000002315.5; Ensembl release 110) using the kallisto software (v.0.48.0) [32]. Kallisto output files were summarized to gene-level using the R package *tximport* (v.1.30.0) [33] and setting the “countsFromAbundance” parameter equal to “lengthScaledTPM”. The annotations were retrieved from Ensembl with the R interface *biomaRt* (v.2.58.2) [34].

### Data analyses

Counts were normalized and converted into log2-counts-per-million (logCPM) using the relative log expression normalization method as implemented in the Bioconductor package *edgeR* (v.4.0.1; [35]). Principal Component Analyses (PCA) were carried out and plotted using the *prcomp* and *ggplot* functions, respectively. Differential expression (DE) analysis between defective meat showing myopathies and normal meat (control) was conducted using the functions *glmFit()* and *glmLRT()* available in the *edgeR* package. Since the number of replicates per condition was low, to enhance the reliability of our findings, we implemented several conservative measures during the DE analysis. Firstly, we employed the filterByExpr() function from the edgeR package to remove low-expressed genes, ensuring robust statistical tests and more accurate fold change calculations for the remaining genes. Secondly, we set an adjusted p-value (False Discovery Rate method) of 0.05 in declaring genes as differentially expressed. Specifically, we set the following contrasts: WS vs. control; WB vs. control; and SM vs. control. Based on the PCA analyses of the whole dataset, the occurrence of sex-specific transcriptional profiles was clearly shown (Supplementary Fig. 1a, Additional File 1). Accordingly, to increase the statistical power and reduce the risk of badly estimating the dispersion of data using the *edgeR* pipeline, two distinct analyses were conducted by separating the male and female datasets.

Functional analyses were conducted in R using *ClusterProfiler* [36]. A Gene Ontology (GO) over-representation analysis was performed through the function *enrichGO()* providing, as gene list, the significant differentially expressed genes (DEGs) resulting from each comparison, and, as background, all the expressed genes. The output was further processed to remove the redundancy of enriched GO terms through the function *simplify()*. A Kyoto Encyclopedia of Genes and Genomes (KEGG) pathway over-representation analysis was also conducted using the function *enrichKEGG()*.

A pre-ranked GO Gene Set Enrichment Analysis (GSEA) was performed to determine whether gene sets defined a priori show statistically significant enrichment at either end of the ranking [37]. A statistically significant enrichment value (Benjamini–Hochberg adjusted p-value ≤ 0.05) indicates that the biological activity (e.g., the biomolecular pathway) characterized by the gene set is correlated with the supplied ranking. The GSEA input was prepared as follows: the raw p-values obtained through the DE analysis conducted by using the *edgeR package* were used to rank the list of genes by significance. To specify the direction of the gene expression variation, the p-values (pval) were replaced by 1-pval or -(1-pval) when a gene was overexpressed or under expressed, respectively, in the defective meat vs. normal meat (control). The GSEA was carried out using both the *gseGO()* and *gseKEGG()* functions.

The *ClusterProfiler* package was then used to produce plots representing enriched terms and gene sets (p-value ≤ 0.05).

## Results

A total of 485,423,575 paired-end reads were sequenced and deposited in GeneBank under BioProject accession PRJNA1154435. These reads were subjected to quality control measures and, after trimming and rRNA removal, almost 21 million reads per sample were retained on average (see Supplementary Table 1, Additional File 1). Approximately 75% of the obtained reads aligned in pairs to the *Gallus gallus* reference genome (see Supplementary Table 1, Additional File 1). The matrix of gene abundances in each sample was then used to carry out the DE analysis in males and females, separately.

### Transcriptional profile

As concerns males, the breast transcriptional profile of broiler chickens with WS, WB, and SM was significantly different from that of those with normal meat, as shown by the PCA (see Supplementary Fig. 1b, Additional File 1). The impact of SM was extraordinarily high; indeed, this myopathy resulted in 4,553 DEGs (2,282 upregulated and 2,271 downregulated genes) compared to normal meat. In male chickens with WB, gene expression changes were moderate, with a total of 386 DEGs (330 upregulated and 56 downregulated) (Additional File 2). Then, weak transcriptional differences were observed in chickens showing WS breast, with only 8 DEGs (5 upregulated and 3 downregulated).

The *edgeR* output of the DE analyses for the three comparisons conducted in males is reported in Additional File 2, whereas the top 20 annotated up- and downregulated genes in each comparison (SM vs. normal, WB vs. normal, and SM vs. normal) are reported in Table 1.

**Table 1:** Top DEGs in male chickens

Ensemble Gene ID	Gene name	lfc	FDR	Ensemble Gene ID	Gene name	lfc	FDR
*SM vs. normal breasts*							
Upregulated genes				Downregulated genes			
***ENSGALG00010010909***	***SLIT3***	3.21	1.96E-12	*ENSGALG00010019820*	*BBOX1*	−2.29	1.96E-12
***ENSGALG00010012939***	***GJA1***	1.82	1.34E-10	***ENSGALG00010012654***	***TENT5B***	−2.64	1.96E-12
*ENSGALG00010019614*	*MICALL2*	1.76	1.34E-10	*ENSGALG00010013457*	*SH3BGR*	−1.88	5.41E-12
*ENSGALG00010010998*	*SOAT1*	1.89	4.24E-10	*ENSGALG00010018322*	*NT5C1A*	−2.03	9.37E-12
***ENSGALG00010018943***	***ACKR4***	3.57	5.48E-10	*ENSGALG00010004312*	*PDLIM5*	−2.12	9.77E-12
*ENSGALG00010013604*	*FYN*	1.42	1.39E-09	*ENSGALG00010017656*	*FSD2*	−2.06	1.56E-11
*ENSGALG00010029936*	*SERPINF2*	2.49	2.94E-09	*ENSGALG00010017115*	*LGALSL*	−2.51	1.95E-11
*ENSGALG00010019572*	*NFKBIE*	1.94	3.02E-09	*ENSGALG00010016224*	*MOSPD1*	−1.89	2.76E-11
*ENSGALG00010013635*	*BCL6*	1.50	3.09E-09	*ENSGALG00010020700*	*MTHFD2*	−2.30	2.80E-11
*ENSGALG00010025982*	*SERPINE2*	2.10	3.21E-09	*ENSGALG00010007532*	*DCUN1D5*	−2.65	5.60E-11
*ENSGALG00010029518*	*FAAP100*	1.68	5.39E-09	*ENSGALG00010024005*	*STYK1*	−2.32	6.37E-11
*ENSGALG00010021045*	*DPP4*	2.26	7.73E-09	*ENSGALG00010000674*	*GRB10*	−1.42	8.06E-11
*ENSGALG00010002281*	*TGFBR2*	1.68	1.45E-08	*ENSGALG00010016374*	*FRMD5*	−2.76	8.30E-11
***ENSGALG00010013954***	***LPL***	1.83	2.25E-08	*ENSGALG00010021619*	*FHOD1*	−1.41	1.97E-10
*ENSGALG00010006446*	*LSAMP*	2.19	2.68E-08	*ENSGALG00010026090*	*USP2*	−1.79	1.97E-10
*ENSGALG00010002389*	*COL21A1*	2.37	2.89E-08	*ENSGALG00010022253*	*ALPK3*	−1.75	2.16E-10
***ENSGALG00010003006***	***CYP7B1***	1.97	3.15E-08	*ENSGALG00010004154*	*COPS5*	−1.95	2.45E-10
*ENSGALG00010002211*	*ACER3*	1.48	3.56E-08	***ENSGALG00010028598***	***TMOD4***	−1.62	3.14E-10
*ENSGALG00010019124*	*BMPER*	2.70	4.43E-08	*ENSGALG00010011578*	*MYLK4*	−4.05	3.14E-10
ENSGALG00010006739	VCAN	2.79	4.54E-08	ENSGALG00010023392	TPI1	−2.59	5.26E-10
*WB vs. normal breasts*							
Upregulated genes				Downregulated genes			
***ENSGALG00010010909***	***SLIT3***	2.25	7.10E-06	*ENSGALG00010015555*	*PNRC2*	−0.86	1.47E-03
*ENSGALG00010027200*	*G0S2*	1.87	2.18E-05	*ENSGALG00010007660*	*FAM98A*	−0.89	5.23E-03
*ENSGALG00010017399*	*CPXM1*	1.62	2.18E-05	***ENSGALG00010012654***	***TENT5B***	−1.06	9.37E-03
***ENSGALG00010012939***	***GJA1***	1.32	2.18E-05	*ENSGALG00010019474*	*TMEM163*	−5.01	1.21E-02
*ENSGALG00010007595*	*ATP2C2*	3.17	1.52E-04	***ENSGALG00010028598***	***TMOD4***	−0.72	1.52E-02
*ENSGALG00010018943*	***ACKR4***	2.48	1.53E-04	*ENSGALG00010022246*	*RHOV*	−4.45	1.56E-02
***ENSGALG00010013954***	***LPL***	1.36	3.09E-04	*ENSGALG00010003115*	*UBE3D*	−1.88	1.67E-02
***ENSGALG00010003006***	***CYP7B1***	1.49	3.70E-04	*ENSGALG00010024654*	*CCDC92*	−1.68	1.71E-02
*ENSGALG00010026560*	*TNNI1*	3.75	4.94E-04	*ENSGALG00010006202*	*SACS*	−0.79	1.81E-02
*ENSGALG00010002998*	*LECT1*	3.16	6.42E-04	*ENSGALG00010024671*	*KCTD20*	−1.21	2.02E-02
*ENSGALG00010005760*	*GPR18*	2.64	6.42E-04	*ENSGALG00010009379*	*RYR1*	−0.69	2.32E-02
*ENSGALG00010005444*	*ACTC1*	2.02	6.42E-04	*ENSGALG00010021574*	*TTLL4*	−0.83	2.54E-02
*ENSGALG00010006134*	*K123*	2.78	6.56E-04	*ENSGALG00010016875*	*SESN2*	−0.97	2.54E-02
*ENSGALG00010004887*	*MYH15*	2.73	9.00E-04	*ENSGALG00010021196*	*ATP6V0A1*	−0.77	2.55E-02
*ENSGALG00010008906*	*DBN1*	1.43	9.01E-04	*ENSGALG00010007214*	*MTHFSD*	−0.94	2.55E-02
***ENSGALG00010022929***	***LRP4***	1.83	1.37E-03	*ENSGALG00010025442*	*ABCF2*	−0.88	2.60E-02
*ENSGALG00010002282*	*TSKU*	1.34	1.37E-03	*ENSGALG00010014151*	*ATP23*	−0.60	2.92E-02
*ENSGALG00010012868*	*CTHRC1*	4.38	1.47E-03	*ENSGALG00010022957*	*SLTM*	−0.73	2.97E-02
*ENSGALG00010029271*	*MYL10*	3.97	1.47E-03	*ENSGALG00010025905*	*CPT1A*	−1.02	3.00E-02
*ENSGALG00010002570*	*ADAM12*	1.46	1.55E-03	*ENSGALG00010022568*	*SCRN3*	−0.83	3.02E-02
*WS vs. normal breasts*							
Upregulated genes				Downregulated genes			
*ENSGALG00010029780*	*TEX14*	4.86	9.63E-08	*ENSGALG00010012260*	*MBD2*	−2.53	5.61E-04
*ENSGALG00010007595*	*ATP2C2*	2.55	3.03E-02	*ENSGALG00010021781*	*RAPGEF4*	−4.74	1.23E-03

Shared DEGs between at least two comparisons are in bold

Top annotated DEGs in male chickens showing spaghetti meat (SM), wooden breast (WB), and white striping (WS) compared with control chickens. *lfc* Log_2_ fold change, *FDR* False discovery rate

Male chickens affected by all three myopathies shared 5 DEGs. Chickens with SM and WB breasts had 345 DEGs in common. In contrast, only three DEGs were shared between chickens with SM and WS conditions (Supplementary Fig. 2, Additional File 1).

Notably, 7 out of the 345 shared DEGs between SM and WB breasts were among the top genes listed in Table 1. These genes are involved in cellular migration (Slit guidance ligand 3- SLIT3, atypical chemokine receptor − 4-ACKR4) and communication (Gap junction alpha-1 protein - GJA1), lipid metabolism (Lipoprotein lipase-LPL, Cytochrome P450 family 7 subfamily B member 1- CYP7B1), and muscle contraction (Tropomodulin 4-TMOD4).

In females, the overall impact of myopathies on breast transcriptome was scanty, as showed by the PCA (Supplementary Fig. 1c, Additional File 1). A total of 2, 19, and 14 DEGs were found in chickens with SM, WB, and WS compared to chickens with normal meat, respectively (Table 2, Additional File 2); moreover, only one DEG was shared between chickens with WB and WS breasts, i.e., laeverin (LVRN).

**Table 2:** Top DEGs in female chickens

Ensemble Gene ID	Gene name	lfc	FDR	Ensemble Gene ID	Gene name	lfc	FDR
*SM vs. normal breasts*							
Upregulated genes				Downregulated genes			
*ENSGALG00010029377*	*NCF1C*	1.74	8.84E-03	ENSGALG00010015960	*CHRNA9*	−2.34	8.84E-03
*WB vs. normal breasts*							
Upregulated genes				Downregulated genes			
*ENSGALG00010017207*	*#NA*	1.65	1.74E-06	*ENSGALG00010025650*	*PPP1R1A*	−2.00	4.83E-03
*ENSGALG00010019027*	*GNLY*	1.66	8.49E-05	*ENSGALG00010004429*	*#NA*	−2.27	1.14E-02
*ENSGALG00010027224*	*RARRES2*	1.32	5.92E-03	*ENSGALG00010010577*	*PBX1*	−0.91	4.03E-02
*ENSGALG00010013380*	*PHGDH*	2.71	5.92E-03	*ENSGALG00010010254*	*WDR41*	−1.46	4.75E-02
*ENSGALG00010005070*	*ALB*	8.05	1.14E-02	*ENSGALG00010026023*	*THY1*	0.82	4.75E-02
*ENSGALG00010017200*	*KCNQ4*	2.22	1.61E-02	*ENSGALG00010029316*	*INTS2*	−1.06	4.88E-02
*ENSGALG00010014944*	*FGB*	4.27	2.22E-02				
*ENSGALG00010012015*	*#NA*	1.21	3.70E-02				
*ENSGALG00010014390*	*SPARC*	1.03	3.70E-02				
***ENSGALG00010008956***	***LVRN***	1.49	4.05E-02				
*ENSGALG00010003131*	*TAP1*	1.01	4.26E-02				
*ENSGALG00010028475*	*AMBP*	4.05	4.66E-02				
*ENSGALG00010016119*	*FETUB*	3.72	4.88E-02				
*WS vs. normal breasts*							
Upregulated genes				Downregulated genes			
***ENSGALG00010008956***	***LVRN***	2.01	2.81E-05	*ENSGALG00010026801*	*FMOD*	−3.17	9.29E-03
*ENSGALG00010023136*	*MDK*	1.78	2.65E-04	*ENSGALG00010002294*	*DAZL*	−6.84	9.29E-03
*ENSGALG00010022468*	*MYBPC3*	1.82	9.29E-03	*ENSGALG00010014104*	*ALDH1A1*	−1.63	1.81E-02
*ENSGALG00010014865*	*TLR2*	1.78	1.87E-02	*ENSGALG00010011539*	*LANCL1*	−0.73	2.29E-02
*ENSGALG00010001230*	*PTX3*	2.38	2.63E-02	*ENSGALG00010001268*	*CBLN2*	−3.21	3.61E-02
*ENSGALG00010002274*	*#NA*	8.71	2.74E-02				
*ENSGALG00010015177*	*TC2N*	1.92	3.10E-02				
*ENSGALG00010022248*	*FIGNL2*	2.24	3.32E-02				
*ENSGALG00010002281*	*TGFBR2*	0.80	9.29E-03				

Shared DEGs between at least two comparisons are in bold

Top annotated DEGs in female chickens showing spaghetti meat (SM), wooden breast (WB), and white striping (WS) compared with chickens with normal breasts. *lfc* log_2_ fold change, *FDR* False discovery rate

### Functional analysis

**Fig. 1:**
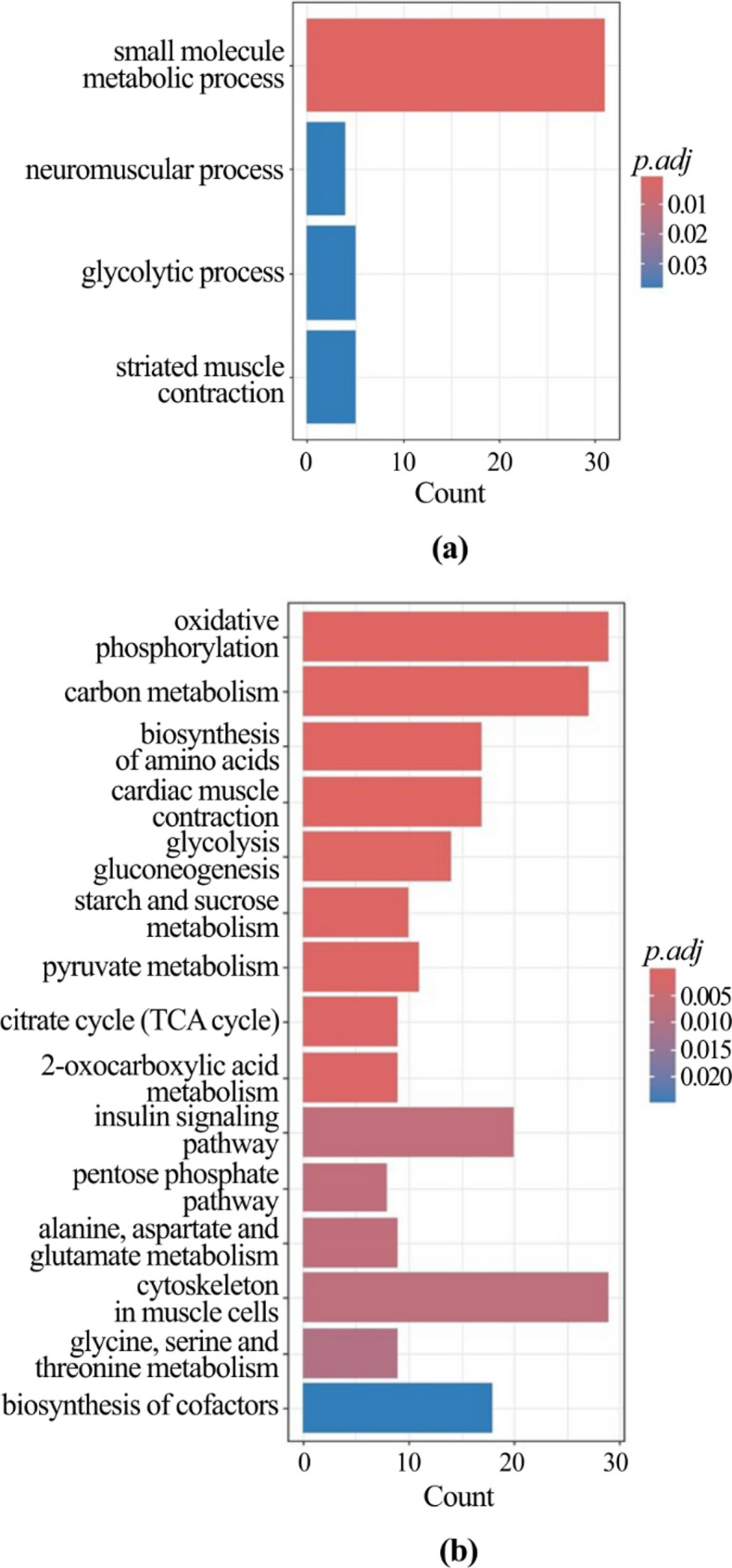
Functional enrichment of downregulated genes in male chickens with spaghetti meat compared with chickens with normal breasts. Bar charts report the over-represented GO terms (**a**) and KEGG pathways **b**. The X-axis reports the number of genes representing each GO term or KEGG pathway. The color gradient corresponds to the significance level adjusted with the false discovery rate method (p.adj)

**Fig. 2:**
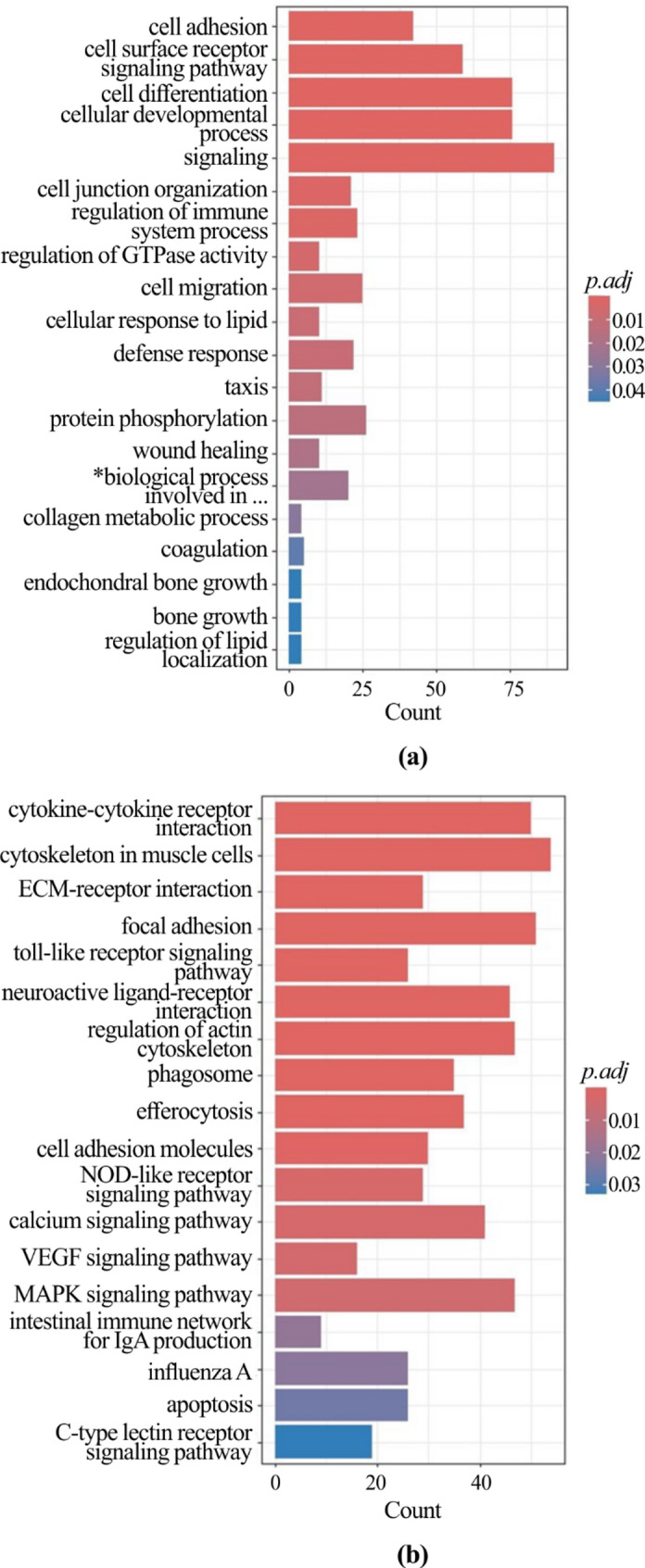
Functional enrichment of upregulated genes in male chickens with spaghetti meat compared with the normal ones. Bar charts report the over-represented GO terms (**a**) and KEGG pathways **b**. The X-axis reports the number of genes representing each GO term or KEGG pathway. The color gradient corresponds to the significance level adjusted with the false discovery rate method (p.adj). *biological process involved in interspecies interaction

The functional interpretation of the lists of DEGs found in chickens with myopathies allowed us to identify the most significant over-represented pathways. As for males (Additional File 3), looking at the downregulated genes in chickens with SM compared to those with normal meat, a total of 4 GO terms and 15 KEGG pathways were significantly over-represented (Fig. 1a and b); as to the upregulated genes, 20 GO terms and 18 KEGG pathways were enriched (Fig. 2a and b). The functional analysis showed that several downregulated genes play a role in muscle contraction and amino acids and glucose metabolism; however, most of upregulated genes are involved in cell signaling and differentiation, and immune processes.

**Fig. 3:**
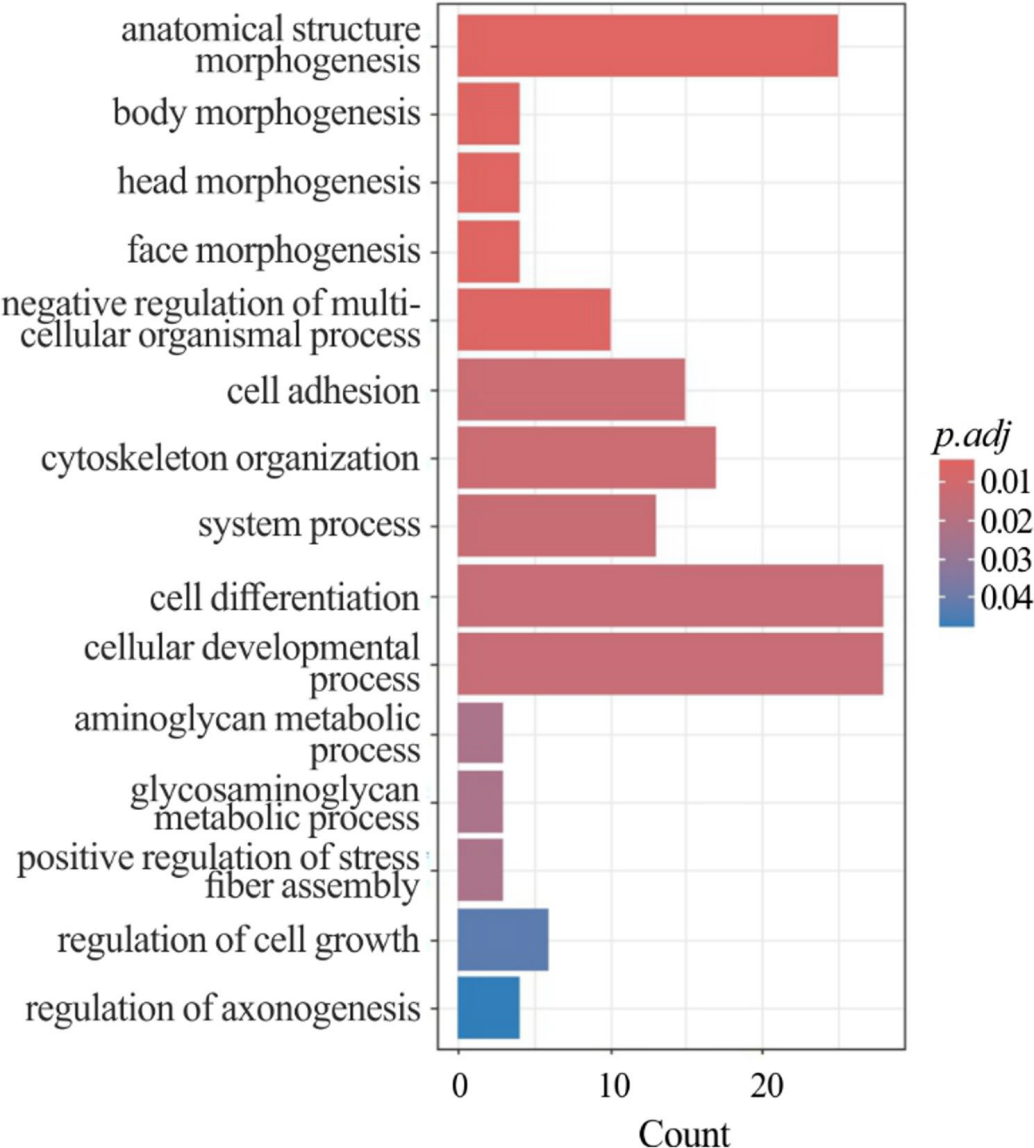
Functional enrichment of DEGs in male chickens with wooden breast compared to the normal ones. The Bar chart reports the over-represented GO terms. The X-axis reports the number of genes representing each GO term. The color gradient corresponds to the significance level adjusted with the false discovery rate method (p.adj)

Since the overall amount of DEGs in male chickens with WB was small, the functional analyses were conducted using all the significantly different expressed genes, both up- and downregulated. This analysis resulted in 15 enriched GO terms, summarized in Fig. 3, and 1 KEGG pathway, namely ‘cytoskeleton in muscle cells’. As a whole, most of genes whose expression changed in WB muscles were involved in cell differentiation, morphogenesis, and glycan metabolism.

**Fig. 4:**
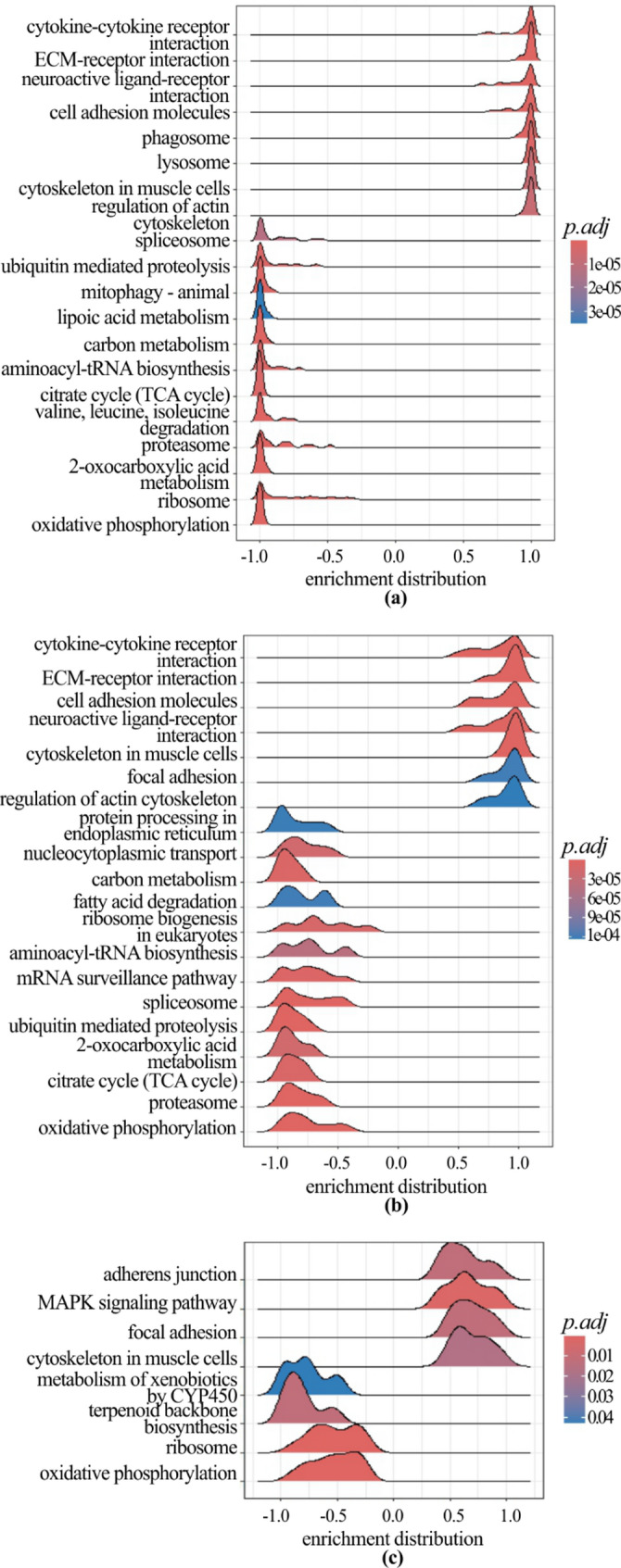
Gene Set Enrichment Analysis in male chickens with myopathies. The ridge plots report the top 20 most significant KEGG pathways activated (enrichment distribution > 0) or suppressed (enrichment distribution < 0) in male chickens with spaghetti meat (**a**), wooden breasts (**b**), and white striping (**c**) compared to normal birds. The color gradient corresponds to the significance level adjusted with the false discovery rate method (p.adj)

The GSEA conducted on all the DEGs in the breasts of male chickens with myopathies resulted in 60 (SM breasts), 50 (WB breasts), and 8 (WS breasts) enriched KEGG pathways compared to males with normal breasts (Additional File 4; Fig. 4). Six pathways were significantly enriched in the breasts of chickens showing myopathies, whatever the specific defect: three of them were activated (‘adherens junction’, ‘focal adhesion’, and ‘cytoskeleton in muscle cells’), while the other three were suppressed (‘oxidative phosphorylation’, ‘ribosome’, and ‘terpenoid backbone biosynthesis’). The breasts of broilers with SM and those showing WB shared 44 enriched KEGG pathways.

With regards to females, the GSEA resulted in 40 (SM breasts), 25 (WB breasts), and 31 (WS breasts) enriched KEGG pathways compared to normal breasts (Additional File 4). Five pathways were significantly enriched in the breasts of chickens showing myopathies, whatever the type of defect, i.e., ‘oxidative phosphorylation’, ‘ribosome’, ‘cytokine-cytokine receptor interaction’, ‘neuroactive ligand-receptor interaction’, and ‘ubiquitin-mediated proteolysis’.

## Discussion

In the last decade, the scientific community has been investigating the breast transcriptional profiles in male chickens affected by myopathies [3, 17, 38]. However, this study is one of the very few ones targeting the sex-differences in transcriptional profiles of breast myopathies.

Despite some limitations, as for the limited number of samples due to a rather low occurrence of affected breasts in the experiment, the overall results from our RNA-seq investigations demonstrated that the breasts of chickens affected by myopathies show distinctive transcriptional profiles, especially in males and in the presence of SM and WB. Conversely, compared to normal breasts, only mild changes were reported in the WS breasts of males and in females whatever the type of myopathy. Accordingly, the present discussion considers separately males and females. Notably, to enhance the reliability of our findings with the available sample size, in the following discussion we will prioritize the most significant DEGs (those with the highest lfc and lowest FDR) and focus on pathway-level analysis, examining groups of functionally related genes, rather than individual ones.

### Transcriptomic profiling of defective breasts in male chickens

In male chickens, the SM myopathy was characterized by the greatest transcriptional changes. Hypoxia and disruptions in energy metabolism and calcium signaling were the key pathways in breasts affected by SM myopathy, potentially contributing to muscle dysfunction; on the other hand, pathways related to the extracellular matrix, tissue remodeling, and effort to restore vascularization through angiogenesis emphasize the muscle attempt to repair and restore homeostasis.

#### Hypoxia, Oxidative Stress and Energy metabolism

As for hypoxia, we recorded the upregulation of hypoxia-inducible factor 1 alpha (*HIF1A*), the main sensor of hypoxia, and the concomitant downregulation of its inhibitor (*HIF1AN*). These changes indicate that the SM-affected breasts were experiencing hypoxic conditions, corroborating previous reports [17, 38, 39]. Mitochondrial dysfunction has been described as a molecular hallmark of response to hypoxic stress [40] and defective avian muscle [41], and such an assumption is consistent with our observation that several genes primarily involved in oxidative phosphorylation (e.g., cytochrome c oxidase - *COX* - subunits 5B, 7B, 6 C, and 7 C; NADH: Ubiquinone oxidoreductase - *NDUF* - subunits A1, A2, A4, A6, A8, A12, B4, S1, S3, S6), citrate cycle (TCA) (e.g., succinate dehydrogenase subunits A - *SDHA* - and B - *SDHB;* isocitrate dehydrogenase 3 subunits alpha - *IDH3A* - and beta - *IDH3B*), and pyruvate metabolism (e.g., Glyoxalase I - *GLO1;* lactate dehydrogenase A - *LDHA*) are downregulated in SM breasts, likely resulting in a reduction of energy production. One of the top 20 downregulated genes in SM breasts of males is triosephosphate isomerase 1 (*TPI1*), a gene involved in the production of pyruvate [42], whose decrease can contribute to muscle pathology. Notably, hypoxia could lead to severe early-onset neurodegeneration [43]. In this regard the *SLIT3* gene, belonging to the axon guidance pathway, was the most upregulated gene in breasts affected by SM and WB; interestingly, its upregulation has been suggested to be highly correlated with myopathies [18].

Then, two notable genes, upregulated in SM and WB breasts and belonging to gap and tight junction (*GJA1* and Molecule interacting with CasL-like 2 - *MICALL2*, respectively), suggest dysregulation of cell communication, as previously reported in 6-week-old chickens with WB [26]. Specifically, the significant upregulation of the *GJA1* gene, encoding for a connexin 43 protein, is likely a cellular response to hypoxia. This aligns with previous studies indicating an increase in *GJA1-20k*, a variant of connexin 43, in response to ischemic injury [44]; besides, GJA1-20k plays a role in the transcriptional regulation of N-cadherin, which influences cell migration [45]. The *GJA1* gene is also positively associated with growth regulation and may serve for future marker-assisted selection of chicken muscle development [46].

In the current study, we observed a downregulation of the glycolysis/gluconeogenesis pathway in SM breasts, as previously seen in WS [38] and WB [28] muscles, corroborating findings on reduced glycogen reserves in affected muscles [47]. In this regard, Lake and Abasht [48] suggested that changes in WB are primarily driven by cellular stress responses triggered by lipid accumulation, leading to oxidative stress and impairment of glycolytic enzymes such as 6-phosphofructo-2-kinase (*PFKFB3*). In fact, a downregulation of *PFKFB3* [38] and *PFKFB1* (present trial) was noticed in SM breasts, which could be partially responsible for the inhibition of glycolysis. Furthermore, in SM breasts we observed a downregulation of insulin receptor substrates 1 and 4 (*IRS1* and *IRS4*, respectively). The former one is a critical mediator of insulin receptor signaling, and we hypothesize this would result in impaired insulin receptor substrate interactions, which are essential processes for initiating downstream signaling pathways controlling energy homeostasis, such as the PI3K/AKT pathway [49].

In SM breasts, the upregulation of peroxisome proliferator-activated receptor gamma (*PPARG*) and the concomitant downregulation of PPARG coactivator 1 alpha (*PPARGC1A*) underscore alterations in mitochondrial biogenesis, lipid metabolism, and lipogenesis [41, 50, 51]. A dysregulation of glycogenolytic enzymes (phosphorylase kinase regulatory - *PHK* - subunits alpha-1, alpha-2, beta, and gamma-1), as previously found in WS breasts [22], indicate an impaired glycogen breakdown. Additionally, the observed downregulation of protein kinase AMP-activated catalytic subunits alpha 2 (*PRKAA2*), beta 2 (*PRKAB2*), and gamma 1 (*PRKAG1*) suggests attenuated cellular energy sensing mechanisms, exacerbating metabolic disturbances and impairing adaptive responses to energy stressors [52].

#### Inflammation and immune response

As for inflammation and immune response pathways, the most upregulated genes in SM breasts of male chickens belonged to the KEGG pathway ‘cytokine–cytokine receptor interaction’. The KEGG pathways ‘Toll-like receptors’ (*TLR*s) and ‘nod-like receptor signaling’, as well as the GO pathway ‘regulation of immune system’ were enriched, indicating an immune disorder and a systemic inflammation response in affected muscle tissue. In addition, several genes connected to these pathways were also upregulated in male chickens showing WB, where endomysial and perivascular inflammatory infiltrates of macrophages and lymphocytes are common microscopical features in all three myopathies [53].

In such a context, different genes for chemokine including C-C motif chemokine ligands (*CCL4*, *CCL5*, *CCL19*, *CCL21*) and chemokine receptors (*CCR5*, *CCR7*, *CCR8*) were upregulated in SM breasts; by contrast, only the *CCL21* gene was upregulated in WB breast. Previous studies suggested that the upregulation of a number of *CCL*s in SM [3] and WB breasts [26] attracts inflammatory cells on the inflammation site and participates in T helper (TH1 and TH2) inflammatory responses. The C-C chemokines are also likely involved in the inflammatory mechanisms underlying myopathies [54]. In this regard, both in SM and WB breasts, we found markedly upregulated the pro-inflammatory interleukin 1 beta (*IL1B*) and its receptor *IL1R2*; by contrast, numerous genes coding for different IL receptors (*IL1R1*,* IL1RAP*,* IL17RA*,* IL17RD*,* IL2RA*,* IL20RA*,* IL21R*,* IL22RA2*,* IL2RG*,* IL5RA*,* IL13RA1*) were upregulated in SM breasts only, which highlights for myopathy-specific differences. Furthermore, the atypical chemokine receptor 4 (*ACKR4*), one of the top 20 upregulated genes we found in SM and WB breasts, reduces chemokine levels and resolves inflammation [55, 56]. The deficiency of this atypical receptor plays a role in fibrosis by regulating IL-6 production and proliferation of cardiac fibroblasts in mice, consequently inhibiting cardiac fibrosis [57]; on the other hand its upregulation, as recorded in the present study, could promote fibrotic changes in affected muscles. Additionally, various cytokines involved in the TGF-β signaling activation, such as the transforming growth factor-beta (TGF-β), IL-1, the Tumor Necrosis Factor alpha (TNF-α) and Platelet-Derived Growth Factors (PDGFs), contribute to the induction of fibrosis but also to wound healing/myofiber repair [54, 58], which simultaneously occur in *p. major* with myopathies. In this regard, the transforming growth factor beta receptor 2 (*TGFBR2*) and the platelet derived-growth factor receptor beta (*PDGFRB*) were upregulated in both SM and WB breasts, while *TGFB3* and *PDGFRA* were upregulated merely in SM breasts.

Another top upregulated gene in SM breasts was the FYN proto-oncogene (*FYN*), which plays a pivotal role in inducing the muscle immunological response that follows denervation in mice [59]. Additionally, the colony stimulating factor 1 gene (*CSF1*) was slightly upregulated in SM and WB breasts. This gene participates in the differentiation, proliferation, and survival of macrophages [60] and its upregulation, associated with inflammatory reaction, has been already recognized as an early sign of WB [61]. Here, it was associated to SM, too.

With regards to *TLRs*, the overexpression of *TLR2* and *TLR4* in WS breasts has been previously reported and it has been suggested they could play a role in early inflammation; indeed, they promote the expression of proinflammatory cytokines in muscle cells, leading to mild inflammation, connective tissue buildup, and fat deposition in damaged muscle tissue. Consistently, in SM breasts we found an upregulation of the *TLRs* (1 A, 2 A, 4, 5, 7, 15) as well as of the myeloid differentiation primary response 88 (*MyD88*) gene, which activates immune cells through TLRs. This latter gene is also upregulated in WB muscles [62]. Notably, in our chickens with WB only *TLR5* was upregulated, thus suggesting a limited activation of inflammatory pathways in male chickens.

#### Calcium signaling

Results of the present study provide evidence of impaired calcium homeostasis and excitation-contraction coupling in SM breasts of male chickens, as previously reported by Che et al. [3] and also in avian WB breasts [41].

Under our conditions, in SM breasts there was a notable downregulation of critical genes involved in calcium release from the sarcoplasmic reticulum (SR), such as ryanodine receptors 1 (*RYR1*) and 3 (*RYR3*), and triadin (*TRDN*), which are all pivotal for coordinating calcium fluxes essential for muscle contraction, whereas *RYR1* was also downregulated in WB breasts, yet at a lower extent compared to SM breasts. In normal skeletal muscles, dihydropyridine receptors on the t-tubule membrane, once activated by neural stimulation, interact with *RYR1* on the SR membrane, causing the release of Ca^2+^ from the SR into the cytosol, and initiating muscle contraction [63]. A severe reduction in *RYR1* expression would likely affect the amount of calcium release, but also impact the stoichiometry of proteins of the calcium-release complex and its function [64]. A ryanodine receptor 2 (*RYR2)* and *RYR3* deficiency has previously been associated with WS meat [17]. Additionally, we found that *TRDN*, coding for a protein (triadin) involved in calsequestrin anchoring at the triad and the *RYR1* function regulation [65], was downregulated in SM breasts. Yet, in our SM breasts, the synaptophysin-like protein 2 (*SYPL2*) gene was also downregulated, as previously found by other authors in WB breasts [61, 66]. This gene is responsible for coding for the M29 protein, which controls calcium release *via* communication between the T-tubule and the junctional SR membrane [67]. Indeed, Welter et al. [66] hypothesized that the downregulation of genes responsible for calcium release from the SR is the mechanism employed to restore calcium homeostasis in the muscles affected by WB.

Moreover, the mild downregulation we observed for ORAI calcium release-activated calcium modulator 1 (*ORAI1*) gene, involved in Store Operated Calcium Entry (SOCE), further underscores disruptions in maintaining physiological SR calcium levels [68]. Any modifications in the SOCE process resulting in disease and mutations in *STIM1* and *ORAI1* have been linked to tubular aggregate myopathy in humans [63]. It is worth noting that ATPase secretory pathway Ca2 + transporting 2 (*ATP2C2*), a gene encoding Ca2+-ATPase SPCA2 (SPCA2) protein, was upregulated not only in SM but also in WB and WS breasts, where SPCA2 expression potentiated Ca^2+^ influx through Orai1 channels in breast cancer-derived cells and human breast tumors [69], suggesting that overexpression of this protein may participate in Ca^2+^ influx responsible for apoptosis in myopathies.

We also observed the upregulation of genes encoding for other plasma membrane channels, including non-selective transient receptor potential channels TRPC (*TRPC6*,* TRPV2*,* TRPM2*,* TRPM8*) and purinergic P2X receptors (*P2RX1*,* P2RX4*,* P2RX5*,* P2RX7*), that regulate Ca^2+^ entry from extracellular space upon cell surface receptor-mediated stimulation [70], which suggest an increased Ca^2+^ influx in SM breasts.

Previous data evidenced excitation-contraction (EC) alterations associated with WB in broiler chickens as early as week 3 of life [61]. In the present study, the downregulation of myosin light chain kinase 2 (*MYLK2)* and 4 (*MYLK4)* (top 20 downregulated genes) in SM and only *MYLK4* in WB could impact muscle contraction in affected breasts, as previously seen in WB [26, 38]. Moreover, a slight downregulation of calmodulins (*CALM2* and *CALM1*), encoding calmodulin isoforms, and an impaired calcium/calmodulin-dependent protein kinase regulation in SM breasts, reduce calcium transport and could affect EC and myogenesis in defective muscles [22, 71]. The observed downregulation of phosphorylase kinase beta (*PHKB*), and troponin C type 1 and 2 (*TNNC1* and *TNNC2*) in SM breasts suggests a decrease in muscle contraction, as previously reported for WS breasts [17].

#### Remodeling of extracellular matrix (ECM)

The remodeling of ECM and fibrosis is one of the main histological features of *p. major* myopathies in broilers. Thus, it is not surprising that in male chickens with SM, the KEGG pathways ‘EMC receptor interaction’ and ‘cell adhesion molecules and focal adhesion’, as well as the GO term ‘cell adhesion and cell migration’ were enriched and upregulated. Likewise, in WB chickens, the GO term ‘cell adhesion’ was enriched.

Accordingly, previous studies evidenced an upregulation of several genes directly involved in the ECM remodeling and tissue fibrosis in *p. major* affected by myopathies, mainly WB [13, 18, 38, 61, 72, 73], with few data about SM [3]. In our trial, genes related to the synthesis of type I and type III collagen (*COL1A1*, *COL3A1*), the most abundant collagen types in skeletal muscles [74] were upregulated in SM breasts, as previously reported only by Che et al. [3]. Furthermore, several genes encoding different types of collagens including other fibrillar collagens (V), fibril-associated collagens (IX, XII, XIV, XVI, XIX), and network-forming collagens (IV, VI) were also moderately upregulated in SM breasts, with collagen type XXI alpha 1 chain (*COL21A1*) being among the top 20 upregulated genes. This latter gene maintains the ECM integrity, but it is also associated with the organization of the ECM components of the blood vessel walls [75]. Interestingly, in previous studies measuring gene expression in WB breasts characterized by severe fibrosis, only *COL3A1* was also upregulated, whereas Pejšková et al. [76] reported *COL3A* and *COL1A1* to be upregulated in *p. major* affected by a severe degree of this myopathy. Additionally, collagen triple helix repeat containing 1 (*CTHRC1*), one of the top 20 upregulated genes in WB and upregulated also in SM muscles, was previously reported in severely damaged skeletal and cardiac muscles of mice with muscular dystrophies [77]. Then, prolyl 4-hydroxylase subunit alpha 3 (*P4HA3*) gene, participating in collagen biosynthesis, was upregulated in SM and WB breasts, although previously demonstrated only in WB muscles [62].

In the present study, decorin was upregulated in both SM and WB breasts of male chickens, whereas Velleman and Clark [78] found that expression of this gene was correlated with WB occurrence. Previous studies [62, 76] also reported the upregulation of several genes of collagen crosslinking enzymes, including lysyl oxidase (*LOX*) in WB myopathy, while results from the present study showed an upregulation of this gene in SM muscles only. Contrarily, the matrix metalloproteinases (MMPs), a family of extracellular zinc- and calcium-dependent endopeptidases primarily involved in the degradation of ECM components including the cross-linked collagen and elastin fibers [73, 79], were largely upregulated in both SM (*MMP2*,* MMP27*,* MMP23B*,* MMP1*,* MMP9*,* MMP10*) and WB (*MMP2*,* MMP27*,* MMP23B*) breasts, as found by other authors in birds affected by WS [80], WB [3, 38, 76], and SM [3]. Besides being related to catabolic activity, MMPs are multifunctional regulators, also involved in the migration and fusion of cells in skeletal muscles. Their upregulation has been previously linked with the inflammatory process in muscular dystrophies and inflammatory myopathies [76, 81]. Thus, the upregulation of different MMPs in SM and WB breasts could be associated with muscle inflammation or/and regeneration [3].

In SM muscles of male chickens, upregulated genes in the cell adhesion molecules enriched pathway have been found to include clusters of differentiation genes (*CD4*,* CD5*,* CD14*,* CD40*,* CD44*,* CD48*,* CD82*,* CD88*), the integrin family (*ITG* subunits *A2*,* A2B*,* A8*,* B2*,* B4*,* B5*,* B8*), laminin family (*LAM* subunits *A3* and *A5*), glycoproteins constituting the basement membrane involved in myoblast proliferation and differentiation [82], and different adhesion genes including neural cell adhesion molecule 1 (*NCAM1*), sidekick cell adhesion molecule 2 (*SDK2*), and cell adhesion molecules (*CADM1*,* CADM3*). Che et al. [3] have also identified *CADM1* and *CADM3* as upregulated genes in chickens with breasts affected by SM and WB, whereas different authors found *ITGB2* and *ITGB7* to be overexpressed in SM and WB muscles [3, 61]. Finally, the great upregulation of thrombospondin 2 (*THBS2*), a pro-fibrotic and anti-angiogenic matricellular protein, in both SM and WB, corroborates previous findings of Che et al. [3] in these two myopathies as well as those of Brothers et al. [13] in male broilers affected by WB. This suggests the upregulation of this gene may inhibit angiogenesis, therefore decreasing oxygen and nutrients supply to skeletal muscle, and resulting in muscle fibrosis [3].

#### Tissue morphogenesis, proliferation and regeneration

The KEGG pathways ‘cytoskeleton in muscle cells’, ‘regulation of actin cytoskeleton’, and ‘neuroactive ligand-receptor interaction’ were enriched in SM breasts, whereas in male chickens with WB the ‘cytoskeleton in muscle cells’ was the sole enriched KEGG pathway. Previous studies reported that regulation of actin cytoskeleton was enriched in *p. major* in 7-week-old broiler chickens with WB [26] and WS [17]. Mutryn et al. [38] suggested that actin cytoskeleton signaling and axonal guidance signaling pathways, which represent a secondary response to muscle tissue damage, had a role in tissue repair in WB.

As for the structural proteins, Soglia et al. [83] hypothesized a strong relationship between desmin (*DES*) and vimentin (*VIM*) expression and regeneration processes in breast-affected myopathies, due to their role in collagen synthesis [84], fibroblast proliferation [85], and stem cells protection during proliferation [86]. These authors reported an upregulation of *VIM* in SM breasts, not supported by significant changes at the protein level. Accordingly, the present study reports the upregulation of both *DES* and *VIM* in *p. major* muscle affected by SM, suggesting that alteration of the expression of these genes could be responsible for disorganized myofibrils, cytoskeletal disruption, and impaired transmission of contractile forces in affected muscles. This finding is supported by the downregulation of the actinin alpha 2 (*ACTN2*) gene in SM breasts, which reduced protein abundance and was previously associated with a weakening Z-disc and cytoskeleton structure in WB muscles [87]. Then, in the present study, tropomodulin 4 (*TMOD4*), exclusive to skeletal muscle fibers, was one of the top 20 downregulated genes in both SM and WB. Tropomodulins are important for muscle cell structure during regeneration due to the regulation of thin filament lengths and activation, and actomyosin cross-bridge formation [88]. The deletion of *TMOD4* in murine models does not lead to myopathy. Normal myofibril organization, thin filament lengths, and actomyosin contractile function in these muscles are maintained due to the compensatory upregulation of tropomodulin 1 (*TMOD1*), which was upregulated in both SM and WB breasts in the present study.

In the context of cell-matrix interactions, genes encoding fibulin 1 (*FBLN1*) and four and a half LIM domains 1 (*FHL1*) were found to be upregulated in both SM and WB breasts, supporting earlier findings in WB [89]. Previous research [90] demonstrated that upregulation of this gene, which regulates muscle hypertrophy by enhancing NFAT activity, has therapeutic potential for skeletal myopathies in a mouse model with Duchenne muscular dystrophy (mdx model). Meanwhile, Marchesi et al. [17] identified *FHL1* as a promising marker for WS detection. Overexpression of *FHL1* promoted the transformation of muscle fibers to the oxidative type [91], which, in the context of the myopathies evaluated in this study, supports fiber switching occurring in *p. major*. Another upregulated gene in SM breasts was cysteine and glycine rich protein 3 (*CSRP3*), which codes for the LIM protein. This protein is crucial for muscle repair and maintenance, as it interacts with proteins like titin and plays a role in intracellular signaling cascades to maintain sarcomere integrity [92]. Additionally, upregulation of *CSRP3* has been associated with altering muscle fiber types from fast to slow, autophagy in muscle cells, and repair mechanism for myofibers, making it a potential molecular marker for myopathies [22, 93, 94]. In this regard, the upregulation of myozenin 2 (*MYOZ2*) and troponin I1 (*TNNI1*) in both SM and WB breasts (top 20 upregulated) suggests a shift from fast to slow muscle fiber types following myofiber damage, as previously reported only in WB breasts [38]. Genes encoding contractile proteins involved in the actin cytoskeleton signaling pathway (myosin light chains - *MYL1*,* MYL10*,* MYL12A*,* MYLK4*, and *MYLK2*) were upregulated in SM breasts in this study. Among them, *MYL10*, which also plays a role in the focal adhesion signaling pathway and may influence skeletal muscle growth and development [95], was one of the top 20 upregulated genes in WB.

Moreover, in the present study a different trend of expression in genes encoding for fibroblasts growth factors (*FGF*) was observed for the first time in SM breasts. In particular, *FGF2*, *FGF7*, and *FGFR3* were upregulated, while *FGF16*, *FGF4*, and *FGFR4* were downregulated. These growth factors play crucial roles in regulating cell growth, proliferation, differentiation, and survival. *FGF2* permits satellite cell proliferation by repressing myogenesis [96], and it plays an important role in angiogenesis; *FGF7* has been found to be involved in wound healing [97]. A different pattern of expression of both growth factors and related receptors involved in muscle repair and regeneration was previously observed in WB [38].

Lastly, several members of the Rho family of GTPases, which regulate the actin cytoskeleton and participate in cell migration and proliferation [38], were differently expressed in SM breasts compared to normal meat in male broilers. Notably, Rho Related BTB Domain Containing 3 *(RHOBTB3*) was greatly downregulated in SM breasts, corroborating previous findings in SM [3] and WB [3, 38]. While Marchesi et al. [17] reported downregulation of Rho associated to coiled-coil containing protein kinase 2 (*ROCK2*) in WS, we found both *ROCK1* and *ROCK2* genes to be downregulated in SM. These genes are involved in the regulation of the cytoskeleton through downstream regulation of actin, myosin, and associated proteins, influencing cell motility and cell adhesion. Additionally, the inhibition of ROCK enhances neurite growth and regeneration and inhibits apoptosis [98, 99]. This could be indicative of the tissue regenerative efforts.

#### Hypoxia, Oxidative Stress and Energy metabolism

The upregulation of the apoptosis pathway found in the present study in male chickens has not been previously described in SM breasts. Indeed, apoptosis could be induced through the caspase family of cysteine proteases [100], where Marchesi et al. [17] found upregulation of the caspase-associated recruitment domain 9 (*CARD9*) gene. In SM breasts, we observed an upregulation of *CARD10* and *CARD11*. The structure of *CARD11* is similar to *CARD9*, suggesting similar functions. Indeed, *CARD11* also binds to the CARD activation domain of B-cell lymphoma/leukemia 10 (*BCL10*) and signals NF-κB activation as *CARD9* [101], suggesting possible involvement in apoptosis. In SM breasts, the upregulation of Fas cell surface death receptor (*FAS*) and Fas ligand (*FASL*) suggests activation of the Fas death receptor-mediated apoptosis pathway.

On the other hand, several genes highly expressed in SM muscle evidence the tissue effort to limit the apoptosis. Namely, upstream caspases (*CASP2*,* CASP8*,* CASP9*), primarily responsible for initiating caspase-activation cascades [100], were upregulated in SM breasts. Likewise other studies found upregulation of the CASP3 and CASP9 in WB [102]. Besides, we observed a muscle tissue effort to inhibit apoptosis. This is highlighted by the upregulation of calpastatin, an endogenous inhibitor of calpain-1 (downregulated in SM) that prevents ER stress [103], as well as the antiapoptotic BCL2 related protein A1 (*BCL2A1*) [104]. Cytochrome c (*CYCS*) mitochondrial apoptogenic factor was also downregulated. Finally, the upregulation of B-cell lymphoma 6 (*BCL6*), one of the top 20 upregulated genes in SM breasts, could be a defense mechanism of the tissue against oxidation; indeed, Kurosu et al. [105] reported that *BCL6* overexpression was found to reduce oxidant-induced apoptosis induced by chemotherapeutic reagents in B-cell lymphoma cells. Overall, these findings illustrate a dynamic process of apoptosis regulation occurring in breast myopathies, reflecting the tissue response to pathological stressors.

#### Angiogenesis

The presence of SM increased the expression of genes associated with Vascular Endothelial Growth Factor (VEGF) and Mitogen-Activated Protein Kinase (MAPK) signaling KEGG pathways linked to angiogenesis in *p. major* muscles. Poor vascularization and reduction of the capillary network following muscle fiber hypertrophy have been proposed among the potential mechanisms of growth-related myopathy, due to the insufficient oxygen supply and metabolic waste products accumulation leading to hypoxia and myodegeneration [20]. Hypoxia in defective muscles is an inducer of *HIF1*, which activates the expression of *VEGFA*, a gene involved in angiogenesis, induction of vascular permeability, and stimulation of cell migration in macrophage lineage and endothelial cells. In addition, several DEGs involved in the MAPK signaling pathway were upregulated in the SM breasts analyzed in the present study. Namely, ras-related C3 botulinum toxin substrate 2 (*RAC2*) downregulation in chickens has been found to lead to the destruction of angiogenesis [106]. Thus, the upregulation of *RAC2* in SM breasts could reflect a stimulation of vessel growth. Genes encoding for Platelet-Derived Growth Factor subunit B (*PDGFB*) and Platelet-Derived Growth Factor Receptor beta (*PDGFRB*), whose signaling is responsible for functional blood vessels establishment [107], were also slightly upregulated in both SM and WB breasts. Finally, the upregulation of genes from the PI3K-Akt signaling pathway is expected to influence the endothelial cells growth by mediating the VEGF [108]. Thus, the upregulation of MAPK and PI3K-Akt signaling genes in SM breasts highlighted the effort of the tissue to restore vascularization under hypoxic conditions. However, the downregulation of VEGFA in defective *p. major* suggests impaired response to angiogenesis stimuli. In agreement, Alnahhas et al. [9] reported the lack of vascularization in *p. major* affected by WS, whereas Zhang et al. [102] reported a reduction in capillaries number per muscle in WB breasts compared to unaffected muscle. Worth noting, in the present study genes encoding for *FGF2* and *SLIT3* emerge as potent inducers of angiogenesis in SM breasts. The gene *FGF2*, upregulated in SM breasts, promotes angiogenesis by inducing the proliferation, migration, and differentiation of many cell types–including endothelial cells, smooth muscle cells, pericytes, and fibroblasts [109], whereas *SLIT3* acts independently of VEGFA to attract endothelial cells *via* Robo4 interaction [110], suggesting diverse mechanisms for vascular remodeling in myopathic tissues. Finally, the upregulation of angiogenic factors in SM breasts, coupled with similar findings in other myopathies, suggests a potential shared aetiology involving impaired vascularization and adaptive responses to hypoxia.

#### Lipid metabolism

The different pattern of expression of genes associated with lipid metabolism confirms lipid accumulation in muscle-affected myopathies. In this regard, the upregulation of lipoprotein lipase (*LPL*) in both SM and WB muscles is related to lipid accumulation [111]. Namely, LPL, an enzyme that catalyzes the hydrolysis of the triglyceride (TG) core of circulating TG-rich lipoproteins, including chylomicrons, low-density lipoproteins, and very low-density lipoproteins, regulates the body disposal of lipids [112]. As a confirmation of our findings, previous studies showed an upregulation of *LPL* in WB [111], but not in WS breasts [113]. The upregulation of *LPL* in *p. major* can be seen as early as 2 and 3 weeks of age [61, 111], as lipid dysregulation occurs before the major macroscopic changes in the muscles [114]. The G0/G1 switch 2 (*G0S2*), the second most upregulated gene in SM breasts was found to inhibit adipose triglyceride lipase (ATGL) activity in mouse and human skeletal muscle and plays a central role in regulating lipid metabolism and substrate oxidation [115], where changes in *G0S2* expression may cause accumulation of lipotoxic species in skeletal muscle, and impair insulin action.

The differences in the expression of sterol O-acyltransferase 1 (*SOAT1*), a gene involved in atherosclerosis, cholesterol content, glucose, and lipid metabolism [116] in WB but mostly in SM breasts compared to normal muscles, further supporting the evidence of changes in lipid and cholesterol homeostasis in broiler chickens affected by myopathies.

### Transcriptomic profiling of defective breasts in female chickens

As a whole, differences between defective and normal meat were less pronounced in females compared to males. To our knowledge, this study is the first to explore the mechanisms behind SM and WS myopathy development in female chickens, while two previous studies investigated WB in female chickens [13, 111].

Concerning SM breasts, the observed upregulation of neutrophil cytosolic factor 1 C (*NCF1C*) is expected to enhance reactive oxygen species (ROS) production through the NADPH oxidase complex [117], thereby influencing immune responses, oxidative stress, and inflammatory pathways in muscle tissue. The sole downregulated gene in SM of female broiler chickens, cholinergic receptor nicotinic alpha 9 subunit (*CHRNA9*), has not yet been related to myopathies, but it was found to be expressed in immune cells and mostly in T cells [118], suggesting its potential role in inflammation and immune response. *CHRNA9* may also affect pluripotency and undifferentiated status of stem cells, by regulating intracellular Ca^2+^ concentrations [119]. Therefore, the possible role of this gene in the regulation of Ca^2+^ influx in affected muscles should be further investigated.

In females with WB, several genes related to inflammation and immune response were dysregulated. The upregulation of granulysin *(GNLY)*, encoding for the cytosolic protein granulysin (expressed in cytotoxic T cells and natural killer cells, and previously associated with polymyositis [120], and chemerin (*RARRES2*), a leukocyte chemoattractant [121], may be related to the inflammation process. Similarly, the upregulation of the transporter 1, ATP binding cassette subfamily b member (*TAP1*), encoding for a protein that, together with TAP2, transports peptides from the cytosol to the endoplasmic reticulum for binding to MHC class I molecules [122], suggests an active immune response in muscle tissue, potentially indicating inflammation. The slight downregulation of the pre-b-cell leukemia homeobox 1 (*PBX1*) gene, encoding for a protein associated with IL10 expression [123], could indicate the possibility of a defective efferocytosis, which is required for inflammation resolution and tissue repair. As to the tissue remodeling, the upregulation of Ca^2+^-binding protein secreted protein acidic and rich in cysteine (*SPARC*) in female WB muscles may play a key role in the pathogenesis of WB, due to its implication in collagen degradation and assembly [73]. Jørgensen et al. [124] found *SPARC* to be an important modulator of the actin cytoskeleton, suggesting a new role of *SPARC* during tissue remodeling. Previous findings showed the higher expression of *SPARC* by regenerating muscle fibers and myoblasts in patients with different types of myopathies in humans [125]. Furthermore, the phosphoglycerate dehydrogenase (*PHGDH*) gene can promote the proliferation and differentiation of chicken myoblasts [126], suggesting that this gene upregulation in WB chickens has a potential role in tissue regeneration. The observed downregulation of protein phosphatase 1 regulatory inhibitor subunit 1 A (*PPP1R1A*), related to carbohydrate metabolism, corroborates the evidence concerning the impaired glucose metabolism found also previously in WB muscles [127].

In WS breasts of females the most significant upregulated gene was *LVRN*. This gene encodes for a metalloprotease involved in the regulation of blood circulation; its expression greatly increases in WS breasts of male chickens [17], in Tibetan chickens adapted to hypoxia [128] and in WB females and SM males in the present study. A similar pattern of expression, i.e., a great upregulation in WS females and in SM and WB males, was highlighted for midkine (*MDK*), previously seen in chickens with WB and WS [17, 18], and underlines an attempt at tissue regeneration. Nihashi et al. [129] suggested that higher levels of MDK protein produced by myogenic cells of UK Chunky chickens may be implicated in muscular inflammation.

Apparently in contrast with previous findings [17], we found a downregulation of *TGFBR2*, which might indicate a disruption in TGF-β signaling, impairing muscle repair and fibrosis processes in WS breasts. Moreover, the cardiac myosin-binding protein C3 gene (*MYBPC3*), encoding for an adrenergic-responsive regulator of cardiac contractility and leading to hypertrophic cardiomyopathy [130], has been found to be upregulated in female WS breasts. Mutations in this gene lead to decreased levels of functional cMyBP-C, resulting in accelerated sarcomere cross-bridge cycling and impaired contractile function in animal models [130]. Then, pentraxin 3 (*PTX3*), a mediator of acute inflammation and innate immunity, was upregulated in WS breasts in our trial, consistently with previous findings on WB and SM breasts [3]. Finally, the observed fibromodulin (*FMOD*) downregulation is expected to alter extracellular matrix and collagen organization, leading to structural abnormalities in WS muscle.

In our experimental conditions, the few differences in gene expression between normal and defective muscles in female birds may be due to the presence of microscopic lesions in muscles that appear normal macroscopically, even though target genes have already begun to reflect the onset of myopathy [114]. This could be particularly true for SM, having in mind that only two genes were differentially expressed compared to the control group.

Brothers et al. [13] suggested the upregulation of genes related to lipid metabolism and accumulation, vascular damage and impaired angiogenesis, as well as oxidative stress responses in males provides insight into the underlying factors that may predispose male broilers to a higher susceptibility to WB compared to females. Accordingly, in the present study, DEGs in male broilers affected by myopathies (mainly SM and WB) belonged exactly to the pathways mentioned above and suspected to confer a higher susceptibility to males. Moreover, the differential response to myopathies at the molecular level between males and females could be the result of complex interactions involving hormonal regulation, genetic differences, and sexual dimorphism. As suggested by Brothers et al. [13], the genetic selection can have produced a more pronounced metabolic shift in males compared to females. For instance, estrogens generally confer protective effects against muscle damage and inflammation in females via their positive effect on satellite cell activation and proliferation [131]. On the other hand, testosterone can enhance muscle hypertrophy [132]. Previous study [133] reported that the transcriptome of normal breast muscle in geese differs between males and females, with sex-specific patterns in gene expression; notably, the authors identified distinct expression profiles in genes related to muscle development and muscle fiber architecture, suggesting that transcripts upregulated in males contribute to the formation of thicker muscle fibers and greater muscle mass. Worth mentioning, male chickens have a significantly higher number of muscle fibers in breast muscles than in female ones [134]. The myopathies’ development has been commonly associated with growth [135], and occurs faster and at higher rates in male than in female chickens, which increases metabolic demands and contributes to muscle hypertrophy, outpacing the development of supporting structures (e.g., vascular and connective tissue). As for WB, in accordance with the present findings, male broilers are likely more prone to oxidative stress than females [13]. Under our conditions, and in agreement with Brothers et al. [13], hypoxia and ischemic stress were more pronounced in males, likely attributable to an inadequate vascular development relative to muscle size. Despite upregulation of angiogenic signals (e.g., VEGF, PDGF, FGF2) found herein, compensatory mechanisms in males may be insufficient or delayed, while female *p. major* showed less angiogenic gene expression disruption. Brothers et al. [13] suggest that, even without being affected by any degree of WB, male broilers are more susceptible to vascular damage and increased antiangiogenic activity than females. Moreover, if hypoxic stress is considered a trigger of myopathies, one possible reason for the increased occurrence of WB in male broilers could be their faster growth and larger body size compared to females, which leads to higher rates of ROS generation without a corresponding increase in antioxidant defense mechanisms to counteract ROS accumulation [13], as found herein. Further, males exhibited stronger activation of immune and inflammatory signaling (e.g., TLRs, cytokine-cytokine receptor interaction, ACKR4, IL1B), indicating more severe and prolonged inflammatory responses than females, which can contribute to fibrosis and degeneration, as shown by upregulation of *COL1A1*, *MMPs*,* THBS2*, and *TGFβ* signaling components in our trial. Finally, male broilers from our trial exhibited strong activation of genes involved in extracellular matrix remodeling, cell adhesion, and fibrosis (e.g., *CTHRC1*,* LOX*,* COL3A1*,* CADMs*, and *MMPs*), indicating more pronounced connective tissue deposition and fibrotic changes compared to females, in whom these pathways were less activated.

## Conclusions

Despite the limitation due to the sample size of some experimental groups, the present study represents the first comprehensive transcriptomic comparison of *pectoralis major* muscles affected by WS, WB, and SM myopathies in male and female broiler chickens. The first findings revealed distinct sex-specific patterns of gene expression and enriched metabolic pathways, with males exhibiting larger differences among meat types compared to females, which highlights the complex interplay of biological mechanisms in myopathy progression. Gender- differences likely arise from the interplay of fast growth and muscle hypertrophy and hyperplasia in males, leading to increased hypoxic stress, oxidative damage, impaired calcium handling, and a stronger inflammatory and fibrotic response, compared to females, where mild transcriptomic disruptions occur. The differential gene expression and pathway enrichment between sexes suggest a notable influence of sexual dimorphism on myopathy pathogenesis. On the other hand, when comparing all three myopathies under the same experimental umbrella, we did notice that SM impaired male transcriptional profiles to a greater extent compared to WB, and especially to WS. In literature, WB and WS myopathies have been investigated more in depth compared to SM, but the present study clearly points to SM as the most complex myopathy, triggering the modifications of several genes and resulting in a balancing of molecular events associated with tissue damage and recovery. Based on functional analyses and top-DEGs, transcriptional modifications occurring in all three myopathies (yet to a different extent), were those related to energy metabolism, tissue morphogenesis and remodeling, calcium influx. In particular, *ATP2C* stands out as a greatly upregulated gene having a role in Ca2 + influx, which in turn triggers apoptosis in myopathies. Therefore its expression should be specifically investigated as a putative marker of muscle pathologies.

Several similarities were noticed only when comparing SM and WB: hallmarks of these myopathies were inflammation, immune response, apoptosis, lipid metabolism, and angiogenesis. Overall, as detailed throughout the discussion, the number of DEGs representing each biological pathway was always higher in SM compared to WB myopathy. Moreover, even when a gene was reported to be significantly different in both myopathies, the extent of its variation compared to normal muscle was always higher in SM compared to WB (i.e., higher lfc).

Future research should explore the precise molecular mechanisms driving sex-related differences, using a larger dataset of samples for transcriptomic analyses, investigating multiple time points, and adding complementary approaches providing further molecular information to be coupled with gene expression (e.g., miRNA expression). Accordingly, it will be crucial to evaluate potential sex-specific mitigation strategies for improving muscle health in broilers.

## Supplementary Information

Supplementary Material 1.

Supplementary Material 2.

Supplementary Material 3.

Supplementary Material 4.

## Data Availability

Raw sequencing data have been deposited in GeneBank under the BioProject Accession number PRJNA1154435. Data is provided within the manuscript or supplementary information files. The other datasets analysed in the current study are available from the corresponding author upon reasonable request.

## Abbreviations

WS: White striping
WB: Wooden breast
SM: Spaghetti meat
logCPM: log2-counts-per-million
rRNAs: Residual ribosomal RNAs
DE: Differential expression
GO: Gene Ontology
DEGs: Differentially expressed genes
KEGG: Kyoto Encyclopedia of Genes and Genomes
GSEA: Gene Set Enrichment Analysis
SLIT3: Slit Guidance Ligand 3
ACKR4: Atypical Chemokine Receptor 4
GJA1: Gap junction alpha-1 protein
LPL: Lipoprotein lipase
CYP7B1: Cytochrome P450 Family 7 Subfamily B Member 1
TMOD4: Tropomodulin 4
LVRN: Laeverin
HIF1A: Hypoxia-inducible factor 1 alpha
HIF1AN: Hypoxia Inducible Factor 1 Subunit Alpha Inhibitor
COX: Cyclooxygenase
NDUFB: NADH:Ubiquinone Oxidoreductase Subunit B
TCA: Citrate cycle
SDH: Succinate dehydrogenase
IDH3A: Isocitrate Dehydrogenase (NAD(+)) 3 Catalytic Subunit Alpha
GLO1: Glyoxalase I
LDHA: Lactate dehydrogenase-A
TPI1: Triosephosphate isomerase 1
MICALL2: Molecule interacting with CasL-like 2
PFKFB3: 6-phosphofructo-2-kinase
IRS: Insulin receptor substrates
PDPK1: 3-phosphoinositide dependent protein kinase 1
AKT2: Kinase upstream of AKT serine/threonine kinase 2
PPARGC1A: PPARG coactivator 1 alpha
ACACA: Acetyl-CoA carboxylase alpha
FASN: Fatty acid synthase
PHKA: Phosphorylase Kinase Regulatory Subunit Alpha
PHKB: Phosphorylase Kinase Regulatory Subunit Beta
PHKG1: Phosphorylase kinase regulatory subunit gamma 1
AMPK: AMP-activated protein kinase
PRKAA2: Protein Kinase AMP-Activated Catalytic Subunit Alpha 2
PRKAB2: Protein Kinase AMP-Activated Catalytic Subunit Beta 2
PRKAG1: Protein Kinase AMP-Activated Catalytic Subunit Gamma 1
TLR: Toll-like receptor
CLL: C-C motif chemokine ligand
CCR: Chemokine receptors
TH: T helper
IL1B: Inflammatory interleukin 1 beta
ILR: IL receptors
ACKR4: Atypical chemokine receptor 4
TGF: β transforming growth factor-beta
TNFα: Tumor necrosis factor alpha
PDGF: Platelet-derived growth factor
TGFBR2: Transforming growth factor beta receptor 2
PDGFRB: Platelet derived-growth factor receptor beta
FYN: FYN proto-oncogene
CSF1: Colony stimulating factor 1 gene
MyD88: Myeloid differentiation primary response 88
SR: Sarcoplasmic reticulum
RYR1: Ryanodine receptor 1
RYR3: Ryanodine receptor 3
TRDN: Triadin
RYR2: Ryanodine receptor 2
ORAI1: Release-activated calcium modulator 1
SOCE: Store Operated Calcium Entry
ATP2C2: ATPase secretory pathway Ca2+ transporting 2
SPCA2: Ca2+-ATPase SPCA2
TRPC: Transient receptor potential channels
P2RX: Purinergic P2X receptors
MYLK2: Myosin light chain kinase 2
MYLK4: Myosin light chain kinase 4
CALM: Calmodulin
EC: Excitation-contraction
PHKB: Phosphorylase kinase beta
TNNC1: Troponin C type 1
TNNC2: Troponin C type 2
ECM: Remodeling of extracellular matrix
COL1A1: Type I collagen
COL3A1: Type III collagen
COL21A1: Collagen type XXI alpha 1 chain
CTHRC1: Collagen triple helix repeat containing 1
P4HA3: Prolyl 4-hydroxylase subunit alpha 3
LOX: Lysyl oxidase
MMPs: Matrix metalloproteinases
CD: Clusters of differentiation genes
ITGA: Integrin alpha subunits
ITGB: Integrin beta subunits
LAMA: Laminin subunits alpha
NCAM1: Neural cell adhesion molecule 1
SDK2: Sidekick cell adhesion molecule 2
CADM: Cell adhesion molecules
THBS2: Thrombospondin 2
DES: Desmin
VIM: Vimentin
TMOD4: Tropomodulin 4
TMOD1: Tropomodulin 1
FBLN1: Fibulin 1
FHL1: Four and a half LIM domain 1
CSRP3: Cysteine and glycine rich protein 3
MYOZ2: Myozenin 2
TNNI1: Troponin I1
MYL: Myosin light chains
FGF: Fibroblasts growth factors
RHOBTB3: Rho Related BTB Domain Containing 3
ROCK: Coiled-coil containing protein kinase
CARD: Caspase-associated recruitment domain
BCL10: B-cell lymphoma/leukemia 10
FAS: Fas cell surface death receptor
FASL: Fas ligand
BAK1: BCL2 antagonist/killer 1
BCL2A1: BCL2 related protein A1
CYCS: Cytochrome c
Bcl-6: B-cell lymphoma 6
VEGF: Vascular Endothelial Growth Factor
MAPK: Mitogen-Activated Protein Kinase
RAC2: Ras-related C3 botulinum toxin substrate 2
PDGFB: Platelet derived growth factor subunit B
PDGFRB: Platelet derived growth factor receptor beta
LPL: Lipoprotein lipase
MGLL: Monoglyceride lipase
G0S2: The G0/G1 switch 2
SOAT1: Sterol O-acyltransferase 1
NCF1C: Neutrophil cytosolic factor 1C
ROS: Reactive oxygen species
CHRNA9: Cholinergic receptor nicotinic alpha 9 subunit
GNLY: Granulysin
RARRES2: Chemerin
TAP1: ATP binding cassette subfamily b member
PBX1: Pre-b-cell leukemia homeobox 1
SPARC: Secreted protein acidic and rich in cysteine
PHGDH: Phosphoglycerate dehydrogenase
THY1: Thy-1 cell surface antigen
PPP1R1A: Protein phosphatase 1 regulatory inhibitor subunit 1A
MDK: Midkine
MYBPC3: Cardiac myosin-binding protein C3 gene
PTX3: Pentraxin 3
FMOD: Fibromodulin
