# Supplementary material for: Comparative transcriptome analysis of pectoralis major muscles affected by white striping, wooden breast and spaghetti meat in male and female broiler chickens
Source: BMC Genomics. 2025 Aug 21;26:768. doi: 10.1186/s12864-025-11963-6 (PMC12369168; doi:10.1186/s12864-025-11963-6)
Supplement: Supplementary file 1 — Supplementary Material 1. [file 12864_2025_11963_MOESM1_ESM.pdf]

Supplementary Table 1. Synthesized and analyzed libraries in the RNA-seq study.

Sample label	Se x	Myopathie s	Number of raw reads	Number of clean reads	Number of pseudoaligne d reads	Pecentage of pseudoaligne d reads
A2	F	Normal	21,932,683	20,966,001	16,514,108	75,29%
A3	F	Normal	21,672,087	20,626,887	16,463,925	75,97%
A4	F	Normal	26,873,802	25,530,497	20,447,302	76,09%
A5	M	Normal	21,002,192	19,933,417	15,802,951	75,24%
A6	M	Normal	20,388,184	19,292,707	15,305,109	75,07%
A7	M	Normal	21,297,772	20,134,455	16,120,751	75,69%
A9	F	WS	22,095,113	20,993,033	16,811,033	76,08%
A11	F	WS	19,905,269	18,937,478	15,094,297	75,83%
A12	F	WS	23,651,118	22,509,612	17,883,513	75,61%
A13	M	WS	23,238,769	22,116,005	17,609,016	75,77%
A15	M	WS	19,949,668	18,958,752	15,119,757	75,79%
A16	M	WS	27,436,222	25,894,322	20,683,362	75,39%
A17	F	WB	20,955,572	20,045,585	15,910,285	75,92%
A18	F	WB	19,915,471	18,868,882	15,088,366	75,76%
A21	M	WB	22,160,911	21,158,908	17,219,295	77,70%
A22	M	WB	21,486,126	20,451,033	16,299,358	75,86%
A23	M	WB	21,752,868	20,656,322	16,588,781	76,26%
A25	F	SM	20,386,976	19,432,105	15,489,646	75,98%
A26	F	SM	20,659,277	19,757,380	15,879,969	76,87%
A28	F	SM	22,674,349	21,489,425	17,239,426	76,03%
A29	M	SM	22,440,896	21,403,649	17,694,310	78,85%
A30	M	SM	23,548,250	22,273,774	18,134,381	77,01%

The table reports for each library the sex of the bird from which *p. major* muscle was collected (F: female; M: male), their myopathy (N: normal, no myopathy; WS: white striping; WB: wooden breast; SM: spaghetti meat), the number of raw reads sequenced, the number of clean reads (i.e., passing the quality-check), the number of reads pseudaligned (using kallisto pseudoaligner) against the reference genome, and the percentage of percentage of pseudoaligned reads.

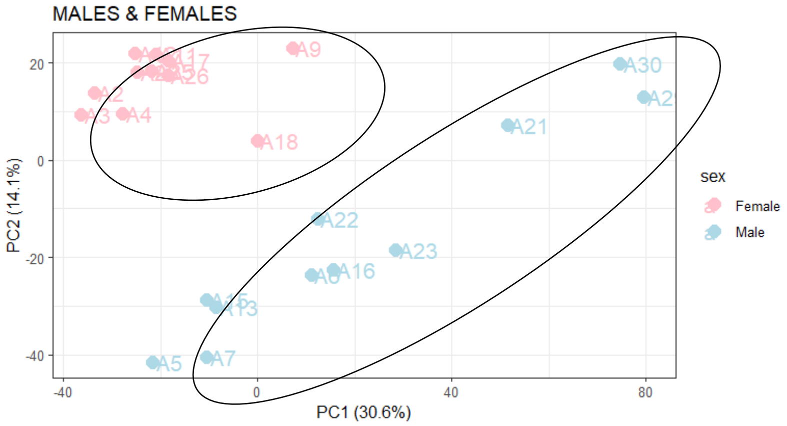

(a)

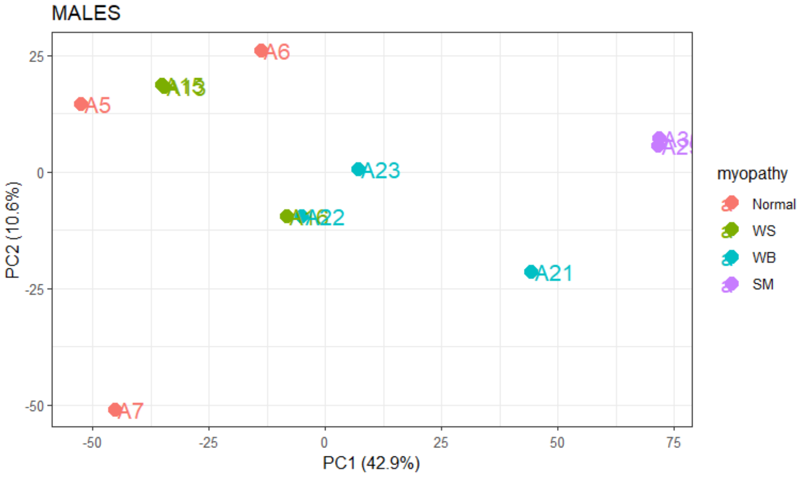

(b)

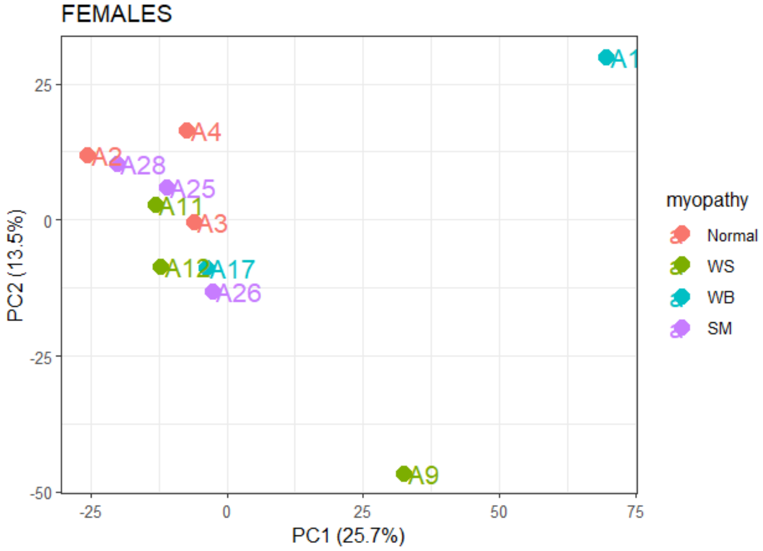

(c)

Supplementary Figure 1. **Principal Component Analysis.** Principal Component Analysis (PCA) conducted on both male and female (a), male (b) and female (c) gene expression profiles. WS: white striping, WB: wooden breast, SM: spaghetti meat, Normal: no myopathies

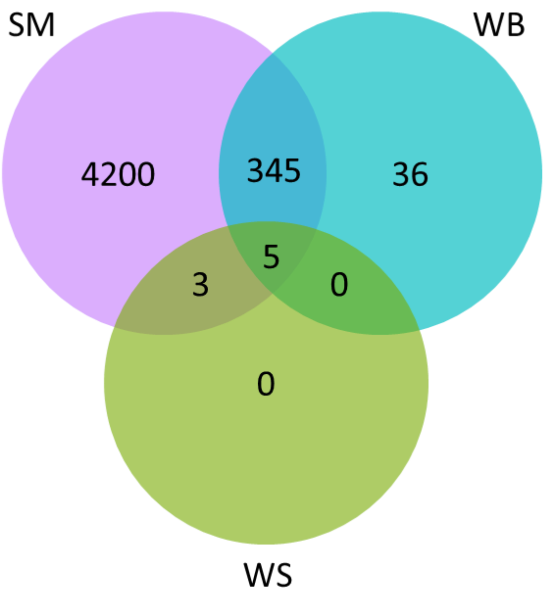

Supplementary Figure 2. **Shared DEGs**. Venn diagram showing shared DEGs between the following pairwise comparisons: white striping (WS) *versus* normal breast, wooden breast (WB) *versus* normal breast, and spaghetti meat (SM) *versus* normal breast.
